# Radiomic model for predicting mutations in the isocitrate dehydrogenase gene in glioblastomas

**DOI:** 10.18632/oncotarget.17585

**Published:** 2017-05-03

**Authors:** Kevin Li-Chun Hsieh, Cheng-Yu Chen, Chung-Ming Lo

**Affiliations:** ^1^ Department of Medical Imaging, Taipei Medical University Hospital, Taipei, Taiwan; ^2^ Research Center of Translational Imaging, College of Medicine, Taipei Medical University, Taipei, Taiwan; ^3^ Department of Radiology, School of Medicine, College of Medicine, Taipei Medical University, Taipei, Taiwan; ^4^ Graduate Institute of Biomedical Informatics, College of Medical Science and Technology, Taipei Medical University, Taipei, Taiwan; ^5^ Clinical Big Data Research Center, Taipei Medical University Hospital, Taipei, Taiwan

**Keywords:** isocitrate dehydrogenase, brain tumor, glioblastoma, computer-aided diagnosis, magnetic resonance imaging

## Abstract

The present study proposed a computer-aided diagnosis system based on radiomic features extracted through magnetic resonance imaging to determine the isocitrate dehydrogenase status in glioblastomas. Magnetic resonance imaging data were obtained from 32 patients with wild-typeisocitrate dehydrogenase and 7 patients with mutant isocitrate dehydrogenase in glioblastomas. Radiomic features, namely morphological, intensity, and textural features, were extracted from the tumor area of each patient. The feature sets were evaluated using a logistic regression classifier to develop a prediction model. The accuracy of the global morphological and intensity features was 51% (20/39) and 59% (23/39), respectively. The textural features describing local patterns yielded an accuracy of 85% (33/39), which is significantly higher than that yielded by the morphological and intensity features. The agreement level (κ) between the prediction results and biopsy-proven pathology was 0.60. The proposed diagnosis system based on radiomic textural features shows promise for application in providing suggestions to radiologists for distinguishing isocitrate dehydrogenase mutations in glioblastomas.

## INTRODUCTION

Glioblastomas (GBMs), the most common glioma, account for approximately 70% of astrocytomas and 15% of all intracranial neoplasms [1, 2]. Approximately 90% of GBMs are classified as primary, and such GBMs mainly affect elderly people, with a median survival of approximately 15 months. The remaining 10% of GBMs are classified as secondary. Secondary GBMs develop from World Health Organization (WHO) grade II or III gliomas and predominantly affect younger individuals, with a median survival of 31 months [3].

The type of astrocytic neoplasm is defined through histological analysis. Recently, exomic sequencing has revealed frequent mutations in the isocitrate dehydrogenase 1 (*IDH1*) gene and its homolog *IDH2*, in both low- and high-grade gliomas [4, 5]. Almost all *IDH1* mutations result in an amino acid substitution at R132. These mutations impair the physiological function of *IDH1* in converting isocitrate to α-ketoglutarate (KG) and confer a gain of function in converting α-KG to D-2-hydroxyglutarate (D-2HG), which accumulates in extremely high levels in tumors with *IDH1* mutations [5, 6]. Such mutations are predominantly observed in secondary GBMs; therefore, these mutations are typically considered as highly selective molecular markers of secondary disease. In the latest WHO tumor classification of the central nervous system (CNS), GBMs are classified as (1) *IDH*—wild-type (WT) and (2) *IDH*-mutant GBMs. However, diagnostic challenges may arise because of the heterogeneity of tumors and partial sampling of lesions. Moreover, not every institute has developed reliable methods to completely evaluate the *IDH* genotype for every case, particularly for cases that cannot be treated through invasive surgery.

Magnetic resonance imaging (MRI) is the modality of choice for imaging brain tumors. It can provide clear tissue contrasts to help in estimating the malignancy of brain tumors [7, 8]. In addition to tumor grading, several physiological MRI techniques, including diffusion-weighted imaging (DWI), MR spectroscopy (MRS), and perfusion-weighted imaging (PWI), have been developed to more accurately characterize the physiological and molecular features of GBMs [9, 10]. Andronesi and Choi [11, 12] have reported that MRI combined with specialized MRS techniques can detect the *in vivo* overexpression of D-2HG. Other techniques, including DWI and PWI, were also applied to detect differences between WT and mutant *IDH* [13]. Tumors with *IDH* mutations were proposed to have a more heterogeneous imaging microenvironment because of their stepwise gliomagenesis [13]. Nevertheless, the relationship between the *IDH* status and tissue signals generated by clinically relevant conventional MR sequences has not been well documented.

For quantifying tumor characteristics through imaging, various computer-aided diagnosis (CAD) systems have been developed for classifying tumor types and grades [14–16]. An artificial intelligence classifier can facilitate combining numerous radiomic features to generate a specific model. In particular, differences between tumors with and those without *IDH* mutations may be subtle and should be explored through the sophisticated integration of various image features. In this study, quantitative morphological, intensity, and textural features were extracted from tumor tissues to determine their *IDH* status; the performance of both individual feature sets and a combination of the 3 feature sets was evaluated. This quantitative CAD procedure can provide consistent and reliable suggestions for *IDH* classifications to radiologists.

## RESULTS

Considering the *IDH* genotype as the diagnostic target, the prediction performance of each feature set was determined (Table 1). The global morphological and intensity features exhibited similar performance: accuracy, 51% (20/39); sensitivity, 57% (4/7); and specificity, 50% (16/32) and accuracy, 59% (23/39); sensitivity, 57% (4/7); and specificity, 59% (19/32), respectively. The textural features describing local patterns in tumors yielded an accuracy of 85% (33/39), a sensitivity of 86% (6/7), and a specificity of 84% (27/32), which are significantly higher than the corresponding values for the morphological and intensity features. The agreement level (*κ*) between the prediction results and biopsy-proven pathology was 0.60. A further experiment for analyzing the combination of the 3 feature sets revealed that only the textural features were selected, with an accuracy of 85%. We selected the features of cluster prominence, cluster shading, maximum probability, difference variance, the information measure of correlation, the inverse difference normalized, and the inverse difference moment.

**Table 1:** Performance of different image feature sets for predicting *IDH* mutations

	Morphology	Intensity	Texture	Texture vs. morphology (*p* value)	Texture vs. intensity (*p* value)
Accuracy	51% (20/39)	59% (23/39)	85% (33/39)	0.0016*	0.0119*
Sensitivity	57% (4/7)	57% (4/7)	86% (6/7)	0.2367	0.2367
Specificity	50% (16/32)	59% (19/32)	84% (27/32)	0.0034*	0.0261*

*p < 0.05 indicates a statistically significant difference.

## DISCUSSION

Immunohistochemistry is a standard method for detecting *IDH* mutations under most clinical scenarios. However, the genetic status within a GBM, including the *IDH* status, shows intratumoral heterogeneity. Performing biopsies of the different parts of tumors may yield different results regarding the *IDH* status. Therefore, another noninvasive method to characterize this information is warranted. Radiomic features are typically extracted from pre-existing MRI images stored in a computer; in practice, this is not an expensive and complicated method. This study revealed that computer-aided radiomic features concerning the microenvironmental texture can distinguish the *IDH1* status of GBMs. Studies [5, 17–19] have reported that *IDH* mutations predominantly occur in secondary rather than primary GBMs. These mutations alter the normal enzyme activity, thus reducing the synthesis of α-KG and NADPH, which makes cells more vulnerable to oxidative stress. Furthermore, these mutations impair the function of *IDH* in converting isocitrate to α-KG and confer a gain of function in converting α-KG to D-2HG, resulting in the overexpression of D-2HG in *IDH*-mutant tumors. The excessive D-2HG is considered an oncogenic metabolite because it induces epigenetic changes that lead to the aberrant regulation of gene expression and perturbed cellular differentiation, which may contribute to tumorigenesis [20, 21]. Furthermore, D-2HG induces increased levels of hypoxia-inducible factor (HIF) -1α. It is a transcription factor that promotes tumor angiogenesis [22], a key element in GBM formation. Because of the stepwise gliomagenesis pattern of secondary GBMs, tumors with these mutations are considered to have a more heterogeneous microenvironment and imaging presentations [13]. Our results revealed that tumors with *IDH* mutations tended to have lower values of inverse difference moment features, an indicator of tissue homogeneity [23], which also suggests more heterogeneous intensities in imaging.

Using a radiomic model for predicting *IDH* mutations provides a connection between intuitive vision and personalized profiling. By applying radiomic image features, tumor characteristics can be quantified without requiring a risky biopsy. In addition, imaging is a routine procedure, with no additional costs. We employed numerous radiomic features in this study for predicting *IDH* mutations. These features were divided into the categories of morphological, intensity, and textural features, which provided different viewpoints for distinguishing GBMs with and without *IDH* mutations.

Table 1 shows that the textural features yielded an accuracy of 85% (33/39), a sensitivity of 86% (6/7), and a specificity of 84% (27/32), which are significantly higher (*p* < 0.05) than the corresponding values for the morphological and intensity features. These results are in agreement with those of a previous study that used textural features to characterize the molecular subtypes of GBM [24]. The difference is that the proposed features and classification method targeted *IDH* mutations, which are recognized as a critical marker in GBM classification. We expect that more correlations between radiomic features and gene expression will be investigated.

**Figure 1:**
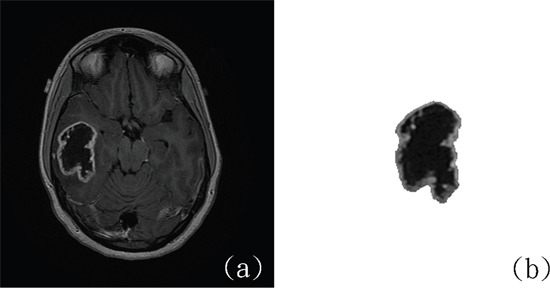
The only *IDH*-mutant case of GBM misclassified using the CAD system **(a)** Original MR image. **(b)** Delineated tumor area. (http://cancerimagingarchive.net/; “License” and the CC BY license, https://creativecommons.org/licenses/by/3.0/; tumor areas in this figure were extracted from original images.)

For predicting *IDH* mutations, we selected the following features: cluster prominence, cluster shading, maximum probability, the information measure of correlation, difference variance, the inverse difference normalized, and the inverse difference moment. The cluster prominence and cluster shading measures indicate whether a lack of symmetry exists in gray-scale distributions. The maximum probability corresponds to the strongest response. The correlation determines the gray-level linear dependence between a pixel and its surrounding neighbors. The difference variance indicates the variance between the co-occurrence probabilities along the x and y axes. The inverse difference moment estimates the homogeneity of the tissue pattern [23]. Regarding the misclassified case in Figure 1, a potential regional feature distinguishing the tissues into numerous regions according to their brightness may be helpful for recognizing the halo observed on the boundary. This type of feature correlated with the regional brightness, and the location can be determined in the future to provide more information than that provided by the existing features.

A limitation of this preliminary study is that the number of *IDH*-mutant cases was not comparable with that of *IDH*-WT cases. Nevertheless, patients in our cohort were enrolled from 4 hospitals, and a balance between the sensitivity (*IDH*) and specificity (non-*IDH*) was maintained (86% and 84%, respectively). Future studies should evaluate more cases with respect to the proposed features and CAD system. Another limitation is that we used only contrast-enhanced T1-weighted images (WIs), which might not clearly demonstrate peritumoral edema. However, *IDH* mutations are associated with angiogenesis activity [22], and the activity of the angiogenesis module within a tumor has been proven to be associated with the degree of contrast enhancement [25, 26]. Therefore, we believe that the measurements of signal intensities on contrast-enhanced T1WIs can be key determinants for differentiating GBMs with and without *IDH* mutations. However, further investigations on the role of other MRI sequences, such as fluid-attenuated inversion recovery, DWI, PWI, and MRS, are warranted.

## MATERIALS AND METHODS

### Patient information

#### The Cancer Genome Atlas and the Cancer Imaging Archive

**Figure 2:**
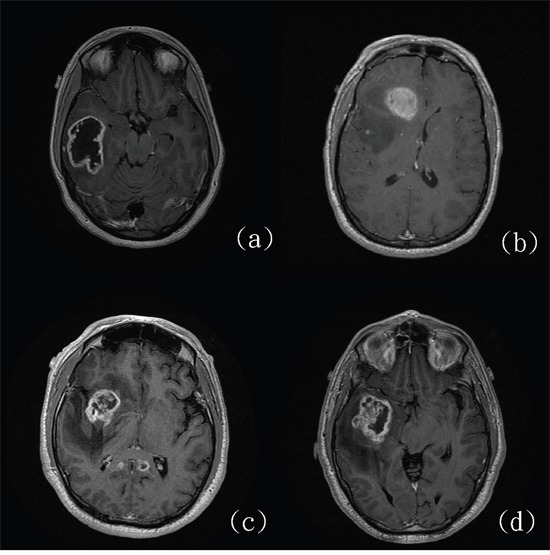
Four glioblastomas with **(a** and **b)** and without **(c** and **d)** *IDH* mutations. (http://cancerimagingarchive.net/; “License” and the CC BY license, https://creativecommons.org/licenses/by/3.0/.)

The MRI data of 32 patients with WT *IDH* and 7 patients with mutant *IDH* in GBM were obtained from the Cancer Imaging Archive (TCIA) (http://cancerimagingarchive.net/) of the National Cancer Institute, a portal that contains images of patients in the Cancer Genome Atlas (TCGA) for performing imaging analysis. The materials and data provided by TCGA were used in compliance with all applicable laws, regulations, and policies for patient protection. All necessary approvals, authorizations, participant assurances, informed consent documents, and institutional review board approvals were obtained [27] (Figure 2).

The MR images used in this study were obtained from different institutes. The *IDH*-WT cases were provided by Henry Ford and Case Western hospitals. The *IDH*-mutant cases were obtained from Henry Ford Hospital, + Case Western Hospital, Emory University, and Fondazione IRCCS Instituto Neuroligico C. Besta. Among the 291 GBM cases in TCIA, only 15 (5.15%) were found to have the *IDH1* mutation. Because 8 of them had no preoperative contrast-enhanced T1WIs, only 7 cases were included in this study. Among them, one case had an *IDH1 R132G* mutation, whereas the remaining cases had an *IDH1 R132H* mutation. No case in TCIA had the *IDH2* mutation. The *IDH*-mutant cases in our cohort were enrolled from 4 hospitals in TCIA. To ensure minimum variation in protocols and equipment, the *IDH*-WT cases were obtained from 2 of the 4 hospitals through consecutive selection from TCGA archive. A total of 40 *IDH*-WT cases were selected; however, 8 cases were excluded because of incomplete or suboptimal image quality. The demographic information of our cohort is listed in Table 2.

**Table 2:** Demographic information of the cohort

	Age	Sex	Tumorlaterality	Tumorlocation
*IDH*-WT	62.6 ± 12.5 years	Female: 9 Male: 23	Right: 20 Left: 12	Frontal: 11 Temporal: 18 Parietal: 2 Occipital: 1
*IDH*-mutant	36.5 ± 15.9 years	Female: 2 Male: 5	Right: 3 Left: 4	Frontal: 3 Temporal: 2 Parietal: 0 Occipital: 2

### Tumor delineation

**Figure 3:**
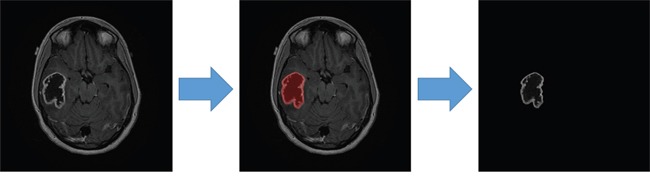
Tumor contour delineation in contrast-enhanced axial T1WIs (http://cancerimagingarchive.net/; “License” and the CC BY license, https://creativecommons.org/licenses/by/3.0/; tumor areas in this figure were extracted from original images.)

Contrast-enhanced axial T1WIs were used for the image analysis. A board-certified neuroradiologist (K.H., having 13 years of experience), blinded to the molecular information, selected the most representative 2-dimensional image of each tumor. Intensity normalization, which extends the gray-level distribution of each MR image to the whole value range (0–255), was performed to enhance the contrast between the tumor and background tissues for contour delineation. The contour was manually delineated using OsiriX. Image pixels enclosed by the defined tumor contour were used for further feature extraction and analysis (Figure 3).

### Radiomic features

**Figure 4:**
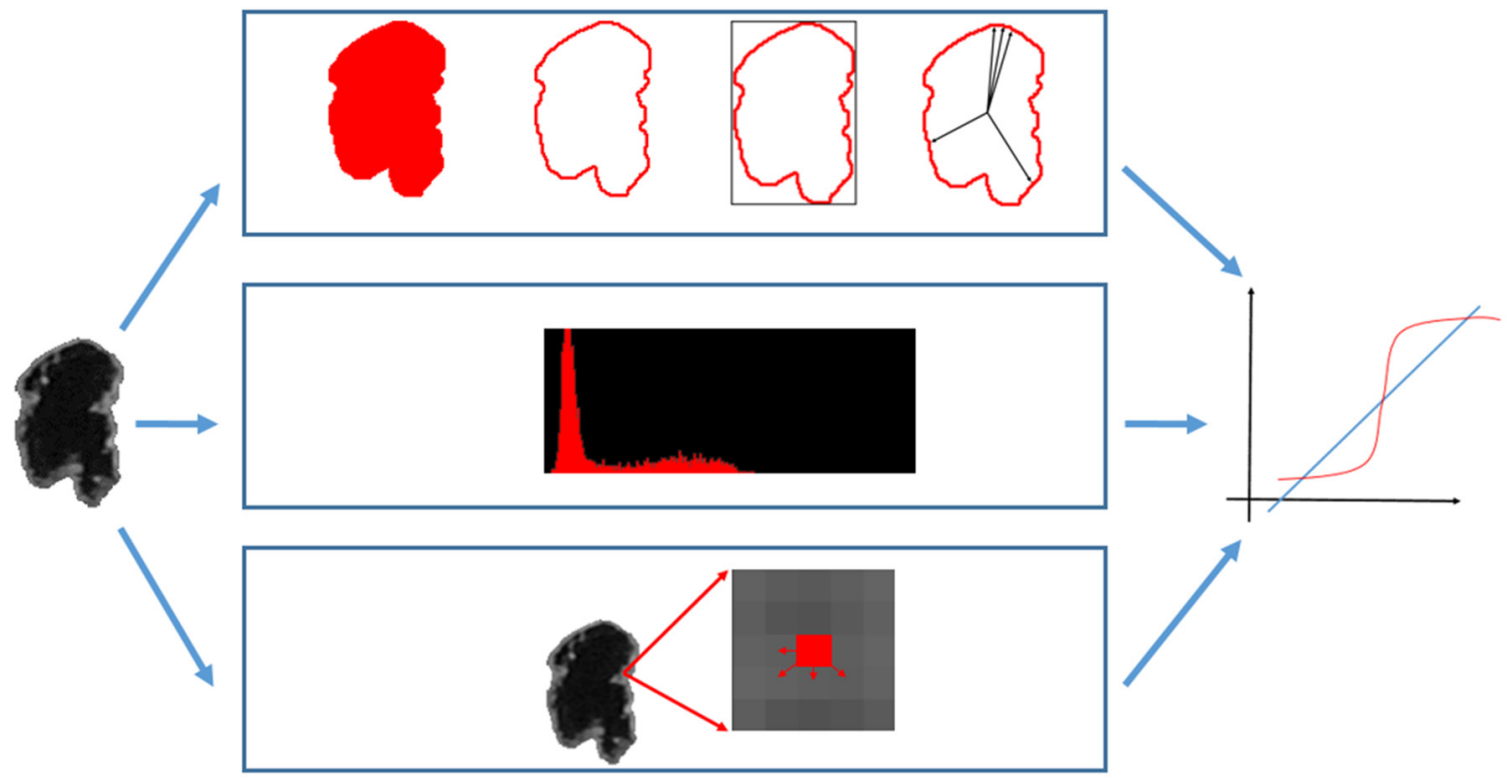
Quantitative features extracted from the tumor area were combined in a logistic regression classifier (http://cancerimagingarchive.net/; “License” and the CC BY license, https://creativecommons.org/licenses/by/3.0/; tumor areas in this figure were extracted from original images.)

The latest WHO tumor classification of the CNS describes *IDH* mutations as a determinant for differentiating GBMs. Whether radiomic features can provide information on *IDH* mutations is relevant to clinical practice. The features extracted from images can be further combined to develop a prediction model for providing suggestions to and improving the diagnostic accuracy of radiologists. Numerous radiomic features were employed in this study for predicting *IDH* mutations. Three categories, namely morphological, intensity, and textural features, were examined to interpret the characteristics of GBMs with *IDH* mutations. Figure 4 illustrates the extraction of radiomic features.

#### Morphological features

A tumor's shape may reflect its growth pattern. Therefore, morphological features are widely used in CAD systems to describe the geometric characteristics of tumors, such as the shape and margins. The essential properties, including the area and perimeter, are easily calculated from the delineated tumor contour. Considering the tumor shape (round or irregular), compactness was proposed to correlate to the tumor area and perimeter [28]. The irregularity of the tumor margin can be estimated using the mean and standard deviation of the normalized radial length (NRL) to determine the complexity of the distribution of the boundary pixels [29]. The NRL is defined as the Euclidean distance between the tumor center and boundary pixels normalized by the maximum distance. The effects of tumors on their adjacent normal tissues may vary. Tumor deformation should be evaluated for tissue characterization.

#### Intensity features

According to the signal intensity characteristics of various tissues in MR images, the brightness distribution reveals the tumor composition. The intensity in contrast-enhanced TIWIs also reveals angiogenesis and blood–tumor barrier abnormalities in brain tumors [9]. By distinguishing the brightness distribution into bins of gray-scale values, a histogram formulates the count of individual values as a probability function. Histogram moments [30, 31] are quantitative metrics for describing probability statistics. Moment features include the first-, second-, third-, and fourth-order central moments of a histogram, namely the mean, variance, skewness, and kurtosis, respectively.

The mean and variance measure the center of the gray-scale value distribution and the extent of increase in these values. Skewness determines the symmetric property of the distribution by describing the balance between 2 sides of a distribution. Kurtosis refers to a single-peak histogram with heavily weighted tails compared with those in a normal distribution.

#### Textural features

Textural features are widely used in CAD systems to discriminate tumor types [15]. A computational statistical analysis between pixels can reveal the local pattern formed by the correlations between adjacent pixels. The brightness composition, such as whether tissues inside the tumor area are heterogeneous or homogeneous, can be determined from textural features.

The gray-level co-occurrence matrix (GLCM) has been proposed as a promising method for interpreting image textures [32–34]. Various CAD systems use GLCM textures to describe image patterns for tumor classification [35]. The GLCM was established by counting the co-occurrence frequencies of 2 adjacent pixels (*i* and *j*) at a distance *d* and direction θ [32]. A reduced image *G* with fewer intensity bins facilitated reducing the computational complexity. Parameters including distance *d* = 1 and 4 directions of θ= 0°, 45°, 90°, and 135° were used individually and in combination. In total, 14 GLCM textural features were used in the experiment:

**Table:** 

*Autocorrelation =*	∑i∑j(px−μx)(py−μy)σxσy;	(1)
*Contrast =*	$\sum_{n}n^{2}\left\{\sum_{i}\sum_{j}p\left(i,j\right)\right\},\left|i-j\right|=n;$	(2)
*Correlation* =	$\frac{\sum_{i}\sum_{j}\left(i-\mu_{x}\right)\left(j-\mu_{y}\right)p\left(i,j\right)}{\sigma_{x}\sigma_{y}};$	(3)
*Cluster prominence* =	$\sum_{i}\sum_{j}{\left(i+j-\mu_{x}-\mu_{y}\right)}^{4}p\left(i,j\right);$	(4)
*Cluster shading* =	$\sum_{i}\sum_{j}{\left(i+j-\mu_{x}-\mu_{y}\right)}^{3}p\left(i,j\right);$	(5)
*Dissimilarity =*	$\sum_{i}\sum_{j}p\left(i,j\right)\left|i-j\right|;$	(6)
*Energy =*	$\sum_{i}\sum_{j}p{\left(i,j\right)}^{2};$	(7)
*Entropy* =	$-\sum_{i}\sum_{j}p\left(i,j\right)\text{log}(p\left(i,j\right));$	(8)
*Homogeneity* =	$-\sum_{i}\sum_{j}\frac{1}{1+i-j}p\left(i,j\right);$	(9)
*Difference variance* =	$\sum_{i}i^{2}p_{x-y}(i);$	(10)
*Difference entropy =*	$-\sum_{i}p_{x+y}\left(i\right)\text{log}(p_{x+y}\left(i\right))$ $\frac{\text{HXY}-\text{HXY}1}{\text{max}\left\{\text{HX},\text{HY}\right\}}$ HXY=(8),;	(11)
*Information measure of correlation =*	$\text{HXY}1=-\sum_{i}\sum_{j}p\left(i,j\right)\text{log}\left(p_{x}\left(i\right)p_{y}\left(j\right)\right)$ $\text{HX}=\text{entropy of }p_{x},$ $\text{HY}=\text{entropy of }p_{y};$	(12)
*Inverse difference normalized =*	$\sum_{i}\sum_{j}\frac{1}{1+\left|i-j\right|}p\left(i,j\right);$	(13)
*Inverse difference moment =*	$\sum_{i}\sum_{j}\frac{1}{1+{\left(i-j\right)}^{2}}p\left(i,j\right);$	(14)

where *μ_x_*,*μ_y_*,_σ*x*_, and σ_*y*_ are the mean and standard deviations of the marginal distributions of *p*(*i, j*|*d, θ*):

$$\mu_{x}=\sum_{i}i\sum_{j}p\left(i,j\right),\mu_{y}=\sum_{j}j\sum_{i}p(i,j)\text{ and}$$ (15)

$$\sigma_{x}^{2}=\sum_{i}{\left(i-u_{x}\right)}^{2}\sum_{j}p\left(i,j\right),\sigma_{y}^{2}=\sum_{j}{\left(j-u_{y}\right)}^{2}\sum_{i}p\left(i,j\right).$$ (16)

### Statistical analysis

The morphological, intensity, and textural image features were implemented in the experiment for tissue characterization. These features completely revealed the global and local appearances of GBMs. The global appearance includes the morphological properties of the tumor shape and overall intensity distribution of tumor tissues, whereas the local texture describes correlations between pixels and their neighbors in different orientations. All features in a category were combined into a feature set in a binary logistic regression classifier for predicting the *IDH* status. A combination of these features was also examined.

With next-generation sequencing-based molecular profiles as the gold standard, imaging features were evaluated using stepwise backward elimination to explore the most favorable combination of features. The feature set with the lowest error rate was considered the most relevant set. The corresponding prediction model was validated using the leave-one-out method [36] to determine its generalizability. In each validation iteration, an individual case was selected and was used to examine the trained prediction model from the remaining *N* − 1 cases. Therefore, each tumor was assigned a probability of having an *IDH* mutation according to its features.

Based on the proven *IDH* genotype, the performance of the prediction model was evaluated using 3 indices: accuracy, sensitivity, and specificity. The chi-squared test was used to compare the performance indices by using SPSS (Version 16 for Windows; SPSS, Chicago, IL, USA). In addition, the prediction result was compared with the biopsy-proven pathology to obtain the agreement level by using Cohen's kappa. The resulting *κ* was −1.0 to 1.0, where a high value indicates high reliability. The agreement was considered slight if *κ* < 0.20; fair if *κ* = 0.21–0.40; moderate if *κ =* 0.41–0.60; substantial if *κ* = 0.61–0.80; and almost perfect if *κ* > 0.81.
